# Protocol for diffusion magnetic resonance imaging and tractography in *ex vivo* mouse models

**DOI:** 10.1016/j.xpro.2026.104667

**Published:** 2026-06-30

**Authors:** Nicholas C. Cottam, Kevin T. Stoll, Jianli Sun, Christine J. Charvet

**Affiliations:** 1 Waisman Center, University of Wisconsin-Madison, Madison, Wisconsin 53705, USA; 2 Idaho College of Osteopathic Medicine, Meridian, Idaho 83642, USA; 3 Department of Biological Sciences, Delaware State University, Dover, DE 19901, USA; 4 Department of Anatomy, Physiology & Pharmacology, College of Veterinary Medicine, Auburn University, Auburn, AL, USA

**Keywords:** Developmental biology, Neuroscience, Systems biology

## Abstract

Diffusion magnetic resonance imaging (dMRI) is a powerful tool to investigate brain structure and connectivity. We present a protocol for quantitative analyses of microstructural properties and white matter pathways via tractography using *ex vivo* dMRI in mouse models. We describe steps for tissue preparation, postmortem brain scanning, diffusion modeling, and tractography. We detail procedures to track white matter trajectories across development and disease states.

For complete details on the use and execution of this protocol, please refer to Cottam et al.^1^

## Highlights

• Steps for collecting *ex vivo* diffusion MRI mouse brain scans
• Instructions for processing diffusion data and generating tractography
• Strategy for integrated analysis of diffusion MRI and tractography with tract-tracers

## Abstract (editor highlights)

Publisher’s note: Undertaking any experimental protocol requires adherence to local institutional guidelines for laboratory safety and ethics.

## Graphical abstract

**Figure:**
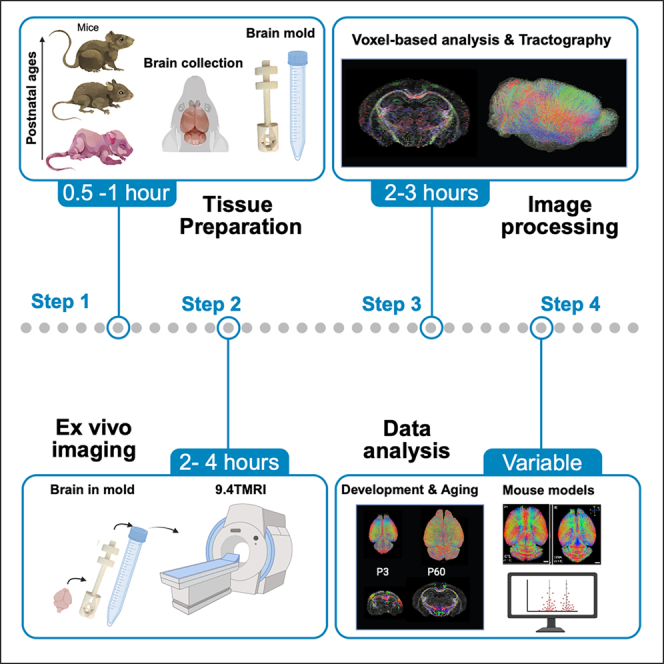

## Before you begin

### Overview

**Figure 1:**
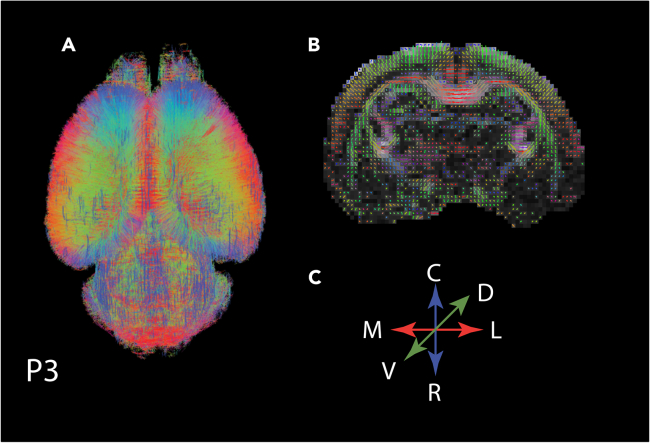
Generation of diffusion MR tractography and metrics We discuss how to use a 9.4 T MR scanner to generate (A) tractographies and (B) diffusivity metrics (e.g., fractional anisotropy). The tractography in this P3 mouse brain shows fibers coursing through its brain. (C) Color coding reflects the (A) average direction of fibers whereas the color coding in (B) shows each voxel’s average diffusion direction. For tractography, fibers coursing across the rostral (R) to caudal (C) direction are blue, those coursing across the medial (M) to lateral (L) direction are red, and those coursing across the dorsal (D) to ventral (V) direction are in green.

Diffusion magnetic resonance imaging (dMRI) is a non-invasive technique that relies on water molecule diffusion to measure microstructural properties.^1^^,^^2^^,^^3^ In the brain, water molecules preferentially diffuse along axons; we can use these properties to study the brain’s structural organization for comparisons across the lifespan and disease. We outline how to collect diffusion MR data from developing mouse brains, which captures the directionality and magnitude of water diffusion within each voxel (Figure 1).

We describe how to scan post-mortem mouse brains, extract diffusivity information to quantify microstructural properties (Figure 1B), and reconstruct brain pathways via tractography. We also provide detailed steps for extracting, fixing, and storing brain samples, performing *ex vivo* MRI scanning in a 9.4 T scanner (Bruker Biospec 94/20), processing the image data, and performing data analyses with both diffusion data and tractography. The protocol is applicable to many cross-sectional and longitudinal studies of development and disease in rodent models.

***Note:*** This protocol is intended for users with direct access to scanning equipment and those relying on core facilities.

This protocol can be used to track maturation of white matter pathways in mice (postnatal day 3 to 60)^4^^,^^5^^,^^6^^,^^7^^,^^8^^,^^9^ and to study mouse models of disease (e.g., spinal muscular atrophy).^10^^,^^11^ We use these as examples throughout the protocol.

Before you begin collecting brain samples or scanning.

1. identify murine ages relevant to human age.^9^^,^^12^
2. Generate brain molds to immobilize post-mortem brains during scanning.
3. Ensure access to a working small animal MRI scanner.

### Innovation

Our protocol integrates high spatial resolution imaging with efficient acquisition times for observing brain structure across murine life stages. These scans generate images with sufficient detail to quantify diffusion properties and reconstruct detailed tractographies at early postnatal stages.^6^^,^^7^ These high spatial resolution diffusion-weighted images can be collected relatively quickly, with a total scan time of approximately three hours. We discuss our workflow for MRI image collection and the usage of software packages to analyze images. Although these methods discussed here were developed for imaging mouse brains, they can be adapted to scan and analyze any small post-mortem tissue.

### Institutional permissions

Animal research first requires approval by the institution’s Institutional Animal Care and Use Committee (IACUC). The collection of brain samples was approved and exempt by several IACUC applications at Delaware State University as specified in past works.^10^^,^^11^ Ensure that you have received proper training to work with post-mortem tissue and rodents (if applicable). Also, ensure that you have received proper safety training to work with an MRI, as there are major safety considerations when working with these scanners.

## Key resources table

**Table:** 

REAGENT or RESOURCE	SOURCE	IDENTIFIER
**Chemicals, peptides, and recombinant protein**		

Fomblin	Solvay Solexis	https://www.solvay.com/en/
Fluorinert	Sigma Aldrich	86508-42-1
4% Paraformaldehyde	Sigma-Aldrich	158127
Phosphate-buffered saline (PBS) 1x	Sigma-Aldrich	P7059
5 mM gadolinium diethylenetriamine pentaacetic acid solution	Sigma-Aldrich	381667

**Software and algorithms**		

Imaging Software	Bruker: ParaVision360	https://www.bruker.com/en/products-and-solutions/preclinical-imaging/paravision-360.html
DSI Studio	Version 2025	https://dsi-studio.labsolver.org/
Trackvis	Version 0.6.1	https://trackvis.org
Diffusion Toolkit	Version 0.6.4	https://trackvis.org
R & R Studio	Posit	https://posit.co/download/rstudio-desktop
Reference brain anatomy atlas	The Allen Brain Atlas	https://mouse.brain-map.org/static/atlas/

**Other**		

3D brain mold	Made from polylactic acid (PLA).	NA
10 ml centrifuge tube	Thermo Scientific™	339650
14 cm straight scissors	Sigma-Aldrich	WPI-501749
Iris scissors	Sigma-Aldrich	WPI-501263
Noyes spring scissors	Sigma-Aldrich	WPI-504491
Plastic pipettes	Sigma-Aldrich	Z331767
Tweezers	Sigma-Aldrich	930229
Stainless steel spatula	Sigma-Aldrich	EISCO-CH0635TFC
Soft bristled brush	Sigma-Aldrich	EISCO-CH0204A
3D printer	ultimaker s5	https://ultimaker.com/materials/s-series-materials/
9.4 T MR scanner	Bruker: BioSpec 94/20	https://www.bruker.com/en/products-and-solutions/preclinical-imaging/mri/biospec.html
MRI RF Surface Coil	Bruker: 1H receive-only 2 x 2 mouse brain surface array coil	https://www.bruker.com/
MRI RF Volume Coil	Bruker: 1H transmit-receive volume coil with active detuning with inner diameter 86 mm	https://www.bruker.com/
Computer Workstation	Macintosh & MainGear Computers	https://www.apple.com/shop/buy-mac; https://maingear.com
Data Storage	Multiple options (e.g., DropBox, Google Drive, External Hardrive)	NA
Display Tablets (Optional)	XP Pen Technology Co Model MD140FH Wacom	DTC121W0 A

## Materials and equipment

### Printing a 3D brain mold

**Figure 2:**
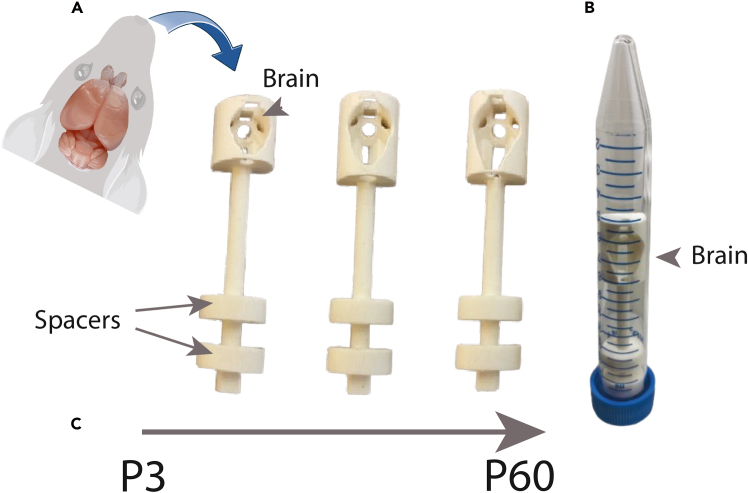
Molds secure the brain in a tube, which can then be submerged with fluid during scanning (A) Here are examples of 3D printed brain molds. We made molds that hold brains of different sizes because mouse brains grow substantially during the first two months of postnatal life age. (B) The 3D brain mold fits into a 10 ml tube.

Subtle movements and exposure to air can compromise image quality (Problem 1). Minimizing brain motion and removing air bubbles are critical for optimal image acquisition. Use a 3D-printed brain molds to submerge the brain in an inert viscous fluid and to secure the brain during scanning (Figure 2). The brain mold contains spacers which securely fit and hold the brain immobile in a 10 mL conical tube (Figure 2B). The spacers and the mold also contain small gaps to allow air bubbles to escape to the surface. We printed molds for mouse brains at approximately postnatal day (P)8, P12, and P60 (Figures 2B and 2C). These molds varied in shape to accommodate mouse brains of different sizes and ages.

***Note:*** The file for the mold is available as Supplementary File 1.

### Generating fixative solutions

We fixed mouse brains through immersion in 4% paraformaldehyde. For 1 L of 4% paraformaldehyde, add 800 mL of 1X PBS to a glass beaker on a stir plate in a ventilated hood. Heat while stirring to approximately 60°C, but the solution should not boil. In a fume hood, add 32 g of paraformaldehyde powder to the heated PBS solution until the paraformaldehyde powder dissolves.

**CRITICAL:** Paraformaldehyde is toxic and you should use appropriate personal protective equipment. Also, heat the materials under a fume hood. The solution should be made fresh for best results.

***Note:*** All relevant institutional protocols must be strictly followed for preparation, use, and disposal of working solutions. Check with your institution about any safety training prior to working with paraformaldehyde. Some institutions have protocols for collecting and degrading working solutions of paraformaldehyde.

### Prepare materials to collect brains

**Figure 3:**
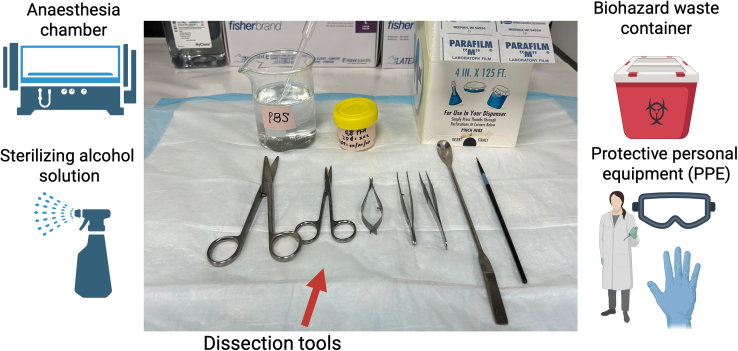
Recommended workstation set up and tools for brain extraction It is ideal to have extraction tools, anesthesia chamber, fixative solution, as well as standard laboratory tools.

Prepare a station and dissection tools to extract and fix mouse brains (Figure 3). Prepare the following materials prior to brain extraction.

• 14 cm straight scissors are used for the decapitation of the head and to generate midline incisions of the head skin in adult mice.
• Iris scissors are used for incisions of adult skull, decapitation of the head, midline incision of the head skin in neonate mice.
• Noyes spring scissors are used for incisions of neonate skull.
• Plastic pipettes are used to rinse the brain and head.
• Tweezers open the skull after incisions.
• Stainless steel spatula removes the brain after opening the skull for adult mice.
• Soft bristled brush work to remove the brain for neonate mice.

### Designing the scan protocol

Complete the safety training for MRI research at our local facility. We acquired diffusion weighted images using spin-echo echo-planar imaging (EPI). This approach provides high scan-time efficiency while enabling the extraction of diverse diffusivity metrics, such as fractional anisotropy. We recommend collaborating with MRI physicists to select the optimal scan protocol (Problem 2). Here are a few considerations to decide on before scanning.

• We recommend collecting structural MR scans (T1 and/or T2 scans) to generate high resolution anatomical images and aid in the localization of myelin content.
• Identify your radio frequency coil and ensure that the specimen will fit within the coil.

***Note:*** We suggest using a 1 H receive-only 2 × 2 mouse brain surface array coil along with a 86 mm active detuned 1 H transmit–receive coil to scan postmortem mouse brains (Bruker). Other coils may be suitable.

For each type of image to be acquired in an MRI protocol, there are multiple parameters to consider:

Magnetic resonance imaging relies on pulse sequence parameters to shape image contrast and quality.^13^ Echo time (TE) is the time interval between the radio pulse and the resulting echo signal, while repetition time (TR) is the interval between consecutive excitation radio frequency pulses. Together, these parameters impact image quality and contrast.

Magnetic diffusion-sensitizing gradients are applied during the scan to collect diffusion weighted images.^1^^,^^2^^,^^3^ The strength and duration of the magnetic diffusion sensitizing gradient is encoded as a *b* value, which we set to 4000 s/mm^2^. Higher *b* values increase sensitivity to tissue microstructure but also result in reduced signal-to-noise ratio, which can lead to erroneous streamline generation during tractography reconstruction. higher-order diffusion modeling that may resolve multiple tissue compartments within a voxel. Therefore, the *b* value balances sensitivity and image quality.

To characterize diffusion along multiple orientations, *b*-vectors are computed for each diffusion-weighted image. These vectors define the specific direction of the diffusion gradients in each acquisition. Together, the *b* values and *b*-vectors form the *b*-table, which encodes the number of diffusion directions and their orientations in three-dimensional space. This table is also used after scanning to reconstruct diffusion models and reconstruct tractographies.

We selected the following scanning parameters.

#### T2-weighted images (scan time = 1 h 15 min)

We used the turbo rapid acquisition with relaxation enhancement (turboRARE) pulse sequence with the following parameters.

• TR/TE = 2500/56 ms
• Number of averages = 2
• 100 μm^3^ isotropic voxel resolution
• rare factor of 14

#### Diffusion-weighted images (scan time = 2 h 10 min)

We acquired diffusion weighted images using a spin-echo echo-planar imaging (EPI) sequence with the following parameters.

• TR/TE = 500/45 ms
• 100 μm^3^ isotropic voxel resolution
• 65 sampling directions
• Single-shell, *b* = 4000 s/mm^2^
• 5 *b* = 0 s/mm^2^ images
• EPI bandwidth = 300,000 Hz
• # of segments = 2
• 3D phase-encoding
• Fat suppression
• Field-of-view saturation

We have optimized our scan parameters and specimen preparation in later experiments. Through consideration with an MRI physicist, we were able to lower scan times without compromising image quality.^11^ We recommend collaborating closely with an MRI physicist to discuss optimal parameters for different tissue types and aims. Single-shot EPI may introduce geometric distortion. Measures should be taken to minimize the effect of geometric distortions from EPI, which can impact output measures. We recommend correcting for background magnetic field inhomogeneities using the field map as a part of the raw outputs and incorporating reverse-phase encoding, which can help preserve geometric fidelity.

### Computing power

Select a computer with sufficient processing power, disk space, and memory to handle image files. An *ex vivo* mouse brain scan requires 5-6 GB of storage. We used DSI Studio, TrackVis, and Diffusion Toolkit software to collect and analyze diffusion MR data (Tables 1 and 2).

• DSI studio is an open-source program that can analyze dMRI images, and which supports different diffusion models (e.g., diffusion tensor imaging; DTI) to reconstruct tractographies.^14^
• Diffusion Toolkit reconstructs tractographies and works with TrackVis to visualize and analyze fiber tracks.^15^ Diffusion Toolkit processes raw DICOM, NiFTI files, and ANALYZE images to generate a Track file that works with TrackVis to display 3D images.

**Table 1:** Recommended hardware used to implement DSI Studio

DSI studio	Recommended Hardware	Hardware used
Chassis:	Dell Precision 7920 Tower	MAINGEAR Turbo Water Cooled
∗Processor:	Two CPUs of Intel Xeon Gold 6230 (2.1GHz, 3.9GHz Turbo, 20 Cores)	AMD Ryzen 7 5800X 8-Core (3.8Ghz)
∗Memory:	128GB RAM	32GB RAM
Graphics Card	NVidia Quadro RTX4000, 8GB	NVidia GeForce RTX3080, 10GB
Hard Drive	2TB SSD	1TB SSD, 4TB HHD
Operating System	Windows	Windows

(∗) denotes key differences between the two hardware specifications

Computer Processing Unit (CPU); Gigahertz (GHz); Gigabyte (GB); Solid State Drive (SSD); Hard Drive (HHD)

**Table 2:** Minimum or recommended hardware to implement Diffusion Toolkit and TrackVis software

Trackvis	Minimum or Recommended Hardware			Hardware
Operating System	Windows 7/8/10	Mac OS X 10.9 or later	Linux	iMac Pro 2017, macOS Mojave (OS X 10.14) 2.3 Ghz Intel Xeon W
∗Memory:	256MB 1-2GB for complex data	256MB 1-2GB for complex data	256MB 1-2GB for complex data	256GB 2666MHz DDR4
Graphics Memory	256MB 512-1024MB recommended	256MB 512-1024MB recommended	256MB 512-1024MB recommended	Radeon Pro Vega 56 8GB
Hard Drive	40MB	120MB	40MB	1TB
Screen Resolution	1280 x 1024 or higher	1280 x 1024 or higher	1280 x 1024 or higher	5120 x 2880

We list the minimum or recommended and the hardware we used for each program (Tables 1 and 2).

## Step-by-step method details

### Sample extraction and collection

**Timing: 1 h + 10 min/sample**

This step describes how to collect, fix and store mouse brain samples.

1. On a multi-layer absorbent pad with plastic backing, prepare the dissection tools.
2. Label a 20 mL plastic container with date and mouse number and fill the container with 10**–**15 mL 4% of paraformaldehyde.
  a. We anesthetize a single animal in a chamber (500 mL volume) with isoflurane inside a fume hood until motion ceases and breathing is stabilized (30**–**60s).
    ***Note:*** Investigators must check with appropriate procedures with their institution about how to collect tissue. Investigators must follow IACUC approved protocols for euthanizing mice of various age ranges. Such protocols are typically established by veterinarians, scientists and the public at each institution. Governing bodies may vary by region.
  b. Remove the animal from the chamber and extract the brain at the workstation. Care should be taken not to damage the brain tissue. Place waste in a container with clear biohazard identification.
    ***Note:*** Transcardiac perfusion was not performed because it can introduce fixation artifacts that impact image quality,^16^ which may be amplified in early-stage P12 mice.
3. To fix the tissue, immerse the brain in 4% formaldehyde within a closable container and seal the container with Parafilm. Store it at 4°C.

***Note:*** Check with your institution to make sure to follow appropriate guidelines for handling parformaldehyde. Paraformaldehyde solution should be sealed in a closed container with the lid manufactured for this purpose given the vessel to be used.

4. Once the brain is saturated (24**–**48 h, typically sinking to the bottom of the container), wash the brain with several changes of 1x phosphate-buffered saline (PBS). We recommend scanning the brain within the next 4**–**6 weeks.
  ***Note:*** We used 0.05% sodium azide to store brains. Sodium azide is classified as a particularly hazardous substance. It is imperative to gain appropriate training to work with sodium azide before use. Exposure to sodium azide may be lethal.
  a. We stored the brain in a PBS solution with 0.02–0.05% sodium azide at 4°C.

### Preparing the mouse brain for scanning

**Timing: 30 min**

This step describes how to prepare use a brain mold so that it the brain is immobile during scanning.

5. 48 hours prior to scanning, wash the brain with several PBS rinses and pat dry.
6. Submerge the brain in 1x PBS and 5 mM gadolinium diethylenetriamine pentaacetic acid solution for 24**–**48 h.
7. Fill a 15 mL conical tube with a chemically inert, viscous, and perflourinated liquid such as Fomblin (alternative Flourinert).

**Note:** Neither of these fluids produce measurable MRI signals and they do not have magnetic susceptibility similar to brain tissue.

8. Carefully coat the brain and brain mold in Fomblin (or Flourinert) and slowly submerge into the test tube. Add more Fomblin (or Flourinert) using a pipette until the tube is completely full, leaving as little air as possible. Overfill with liquid and cap tube.
9. It is critical that there are no air bubbles surrounding the brain. Gently agitate and rotate the tube to guide any air bubbles to the top of the tube (Problem 1). Mix the solution within the tube on a nutating mixer for 1**–**2 h to assist in bubble removal.
10. Wipe the conical tube dry and mark a line on the tube along the midline of the brain to facilitate placement of the brain in the scanner.

### Scanner preparation

**Timing: 15 min**

We describe how to set up the scanner fix the brain surrounded by the coil to collect post-mortem diffusion MR scans.

**Figure 4:**
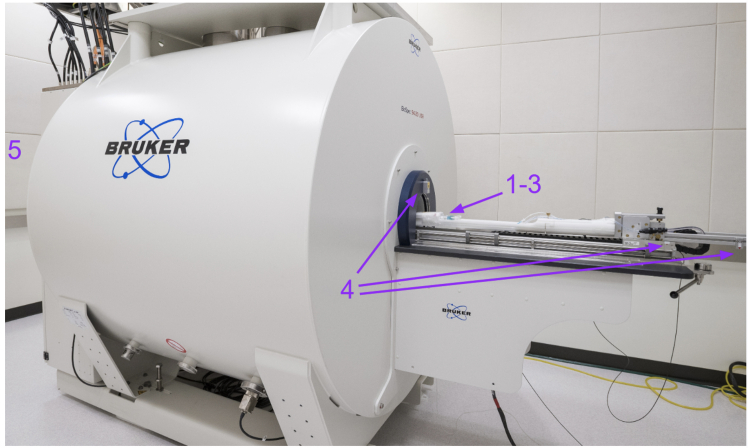
Bruker BioSpec 9.4T MRI scanner at the University of Delaware Each step involved in setting up the scanner is labelled in the text. Image source: https://cbbi.udel.edu/.

Note: We suggest using the ^1^H receive-only 2x2 mouse brain surface array coil with an 86 mm active detuned ^1^H transmit-receive coil.

11. Attach the study-appropriate radio frequency coil to the MRI scanner (Figure 4).

12. Center conical tube within the gradient coil using the mark (Figure 4, step 6; preparing the mouse brain for scanning) and secure the brain tube in place using tape.
13. Secure the gradient coil on the track.
14. Center the gradient coil within the scanner using mounted calibration tools (Problem 3 **and** 4).
15. Tune the scanner after the sample has been placed in the coil.
16. Securely close doors to the scanner room.

### *Ex vivo* magnetic resonance imaging

**Timing: 3–4 h (pre-optimized protocol)**

We outline steps to checks the brain is well positioned before scanning the brain as well as steps to scan the mouse brain.

***Note:*** Describe a scan protocol implemented with the Paravision 2.0 software. Field of view alignment should be considered throughout these steps. We recommend consulting the MR technician for this.

17. Generate a new study if it is the first sample. Assign a sample name to your current scan and create a new scan. Ensure you select the head position as supine.
18. Perform the following scan protocol:
  a. Localizer Plane
    i. Contains a wobble test to make sure that the machine is properly calibrated and balanced prior to scanning.
    ii. Ensures that the brain is properly aligned within the scanner field of view.
    iii. Adjust as necessary and repeat until the brain is in position.
  b. Bubble check
    i. Typically performed using a T1 image protocol. Bubbles are visible and generate image artifacts. In mice, bubbles may appear around the cerebellar cortex and rostral to the pons. Remove bubbles, if any (Problem 1).
  c. Shimming
    i. Makes the magnetic field more uniform within the field of view, which improves image quality.
    ii. Acquire the *b*_*0*_ map (and *b* table)
  d. Diffusion weighted image (DWI) - test
    i. Ensure that the slice package (i.e., the group of individual image slices that are acquired during a radiofrequency pulse) is properly aligned using an abridged imaging step.
    ii. Adjust all of the field of view directions to optimize image saturation.
    iii. Set parameters as desired; these will be copied to the actual diffusion weighted imaging step.
  e. DWI quality control check
    i. Check the signal to noise ratio of the DWI image. Adjust the image contrast as necessary and use region selections to observe the mean signal to noise ratio throughout the scan. A ratio above 8 is the minimum recommendation. If DWI signal-to-noise ratio is consistently below 8, consider decreasing *b* value or echo time.
  f. 3D DWI (2**–**3 h)
    i. Copy the parameters from the test step to this step. These are the diffusion images that will be analyzed in later steps.
  g. 3D T2 image (30**–**60 min)
    i. The images that will be used for segmentation, volumetrics, and anatomical reference.
19. Collect files
  h. Each step from the protocol contains a series of image files.

***Note:*** We recommend exporting both the NifTI and DICOM files to a large-capacity external hard drive or passport for storage. Name your files appropriately (Problem 5).

### Model fitting and tractography

**Timing: 15–30 min per scan**

We describe how to analyze diffusion MR scans. In the following sections, we describe how to use transform the scan data files (NiFTI and/or DICOM) to extract diffusion MR metrics (e.g., fractional anisotropy (FA), mean diffusivity (MD), axial diffusivity (AD), and radial diffusivity (RD)^14^^,^^17^) and perform tractography. We discuss how we use DSI Studio to reconstruct diffusion metrics from.fib files, and how we used Diffusion Toolkit and trackvis to reconstruct and quantify streamlines.^15^

***Note:*** For tractography, DSI Studio processing can also utilize other imaging models such as q-ball imaging (QBI), diffusion spectrum imaging, and generalized q-sampling imaging (GQI), but we focus on using the software to generate diffusivity metric images of the brain from the DTI model.

20. Generating diffusivity metrics with DSI Studio
  The DSI Studio processing pipeline generates images containing DTI metric data (FA, MD, AD, RD). DSI Studio processing steps, masking options, and tractography parameters are described in detail as documentation at dsi-studio.labsolver.org. We provide a brief step-by-step guide for diffusivity metric analyses with DSI studio.
  a. Import 2dseq file and verify the *b*-table, which will generate the.src file (Problem 6).
  b. Reconstruct the file and confirm mask coverage, which will generate the.fib file.
  c. Generate the fiber tracking visualization and select regions of interest.
    ***Note:*** DSI Studio first generates a source (.src) file, which contains the raw diffusion data that is fit to a tensor. This generates NiFTI (.nii) image files for a series of outputs. These include the raw eigenvalues, *b*_*0*_ map, orientation diffusion function and each DTI metric. Fiber tracking and tractography outputs are also reconstructed in the form of a.fib file, which contains a series of coordinates for each streamline.
21. Tractography reconstruction with Diffusion Toolkit

**Figure 5:**
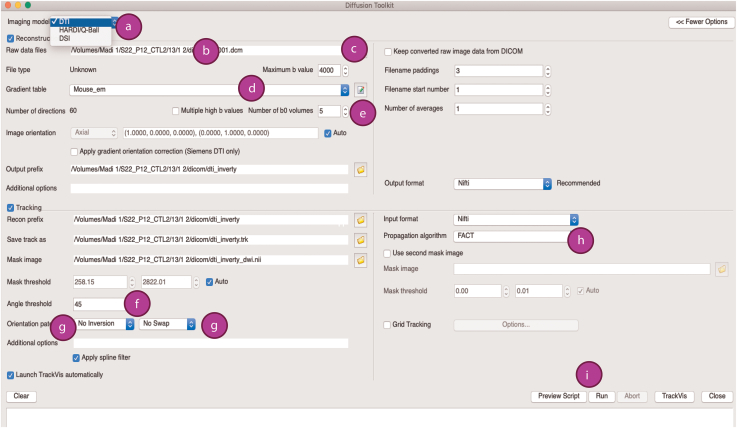
Multiple parameters may impact reconstructed tractographies in Diffusion Toolkit Steps numbered in this figure correspond to steps in the text. Abbreviations: DTI diffusion tensor imaging; HARDI: high angular resolution diffusion imaging; DSI: diffusion spectrum imaging.

We use the Diffusion Toolkit software to perform tractography (Figure 5).^15^ The software provides three options for single-shell diffusion modeling, which is an indispensable processing step for estimating fiber orientations. The software may reconstruct streamlines based on DTI, high angular resolution diffusion imaging (HARDI) and diffusion spectrum imaging.^17^^,^^18^^,^^19^ DTI assumes there is a single fiber orientation within a voxel, and the output measures (FA, MD, AD, and RD) capture different aspects of microstructure. HARDI and diffusion spectrum imaging assume multiple diffusion directions within a voxel and are theoretically adept at distinguishing complex fiber trajectories that cross, kiss, or fan.

***Note:*** Diffusion Toolkit for tractography does not select fibers based on FA threshold. These models require longer scan times and higher *b*-values, which are more susceptible to lower signal-to-noise ratios.

The primary factor determining the selection of an imaging model for your study depends on the *b*-value and number of gradient directions of the MRI scan data. If the *b*-value is around 1000 s/mm^2^ and there are 30**–**65 directions, DTI may be the optimal selection. With a *b*-value between 2500**–**3000 s/mm^2^ and at least 65 directions, the data is adequate for HARDI modelling.^19^^,^^20^^,^^21^^,^^22^ Diffusion spectrum imaging typically requires a *b*-value of 10,000+ s/mm^2^ and 250+ gradient directions, making it the least likely to be applicable to datasets with common acquisition parameters.^15^

***Note:*** In our study, DWIs had a *b-*value of 4,000 s/mm^2^, and thus HARDI and diffusion spectrum imaging were not used to model the data.

22. In Diffusion Toolkit (see Figure 5):
  a. Select the imaging model (DTI, HARDI, or diffusion spectrum imaging) based on your scan parameters (*b*-value and number of gradient directions).
  b. Load your raw data files. The diffusion toolkit can process DICOM, NiFTI, and ANALYZE files.
  c. Input the maximum *b*-value from the images. Here, we used b=4,000s/mm^2^.
  d. Import the *b* table which lists gradient directions for each diffusion-weighted image.
    i. This is typically output to a methods file after scanning.
  e. Input the number of *b*_*0*_ volumes. We used 5 *b*_*0*_ volumes.
  f. Set the angle threshold, which defines the maximum angle of fibers between two voxels. We recommend varying this threshold to evaluate how the angular threshold impacts tractography reconstruction. We used a 45-degree threshold.
  g. Select your orientation patch, which corrects for differences in image orientation interpretation across scanner manufacturers. If the data was acquired on a Siemens scanner, it typically does not need any orientation patches. Bruker, GE and Philips data typically need “Invert Y″, though it is best to test different inversions and swaps (Problem 7).
  h. Select the propagation algorithm to reconstruct fibers. Here, we use the Fiber Assignment by Continuous Tracking (FACT) algorithm for streamline reconstructions.
  i. Click run to start the reconstruction.

***Note:*** We recommended testing multiple inversions and swaps to ensure the appropriate image orientation is selected (Problem 7).

***Note:*** The output will consist of multiple files, including a trackvis file (.trk), which encodes streamlines. The software will also output a *b*_*0*_ NiFTI file that can be used to visualize underlying brain anatomy with streamlines. The.trk file is specific to Trackvis.

## Expected outcomes

**Figure 6:**
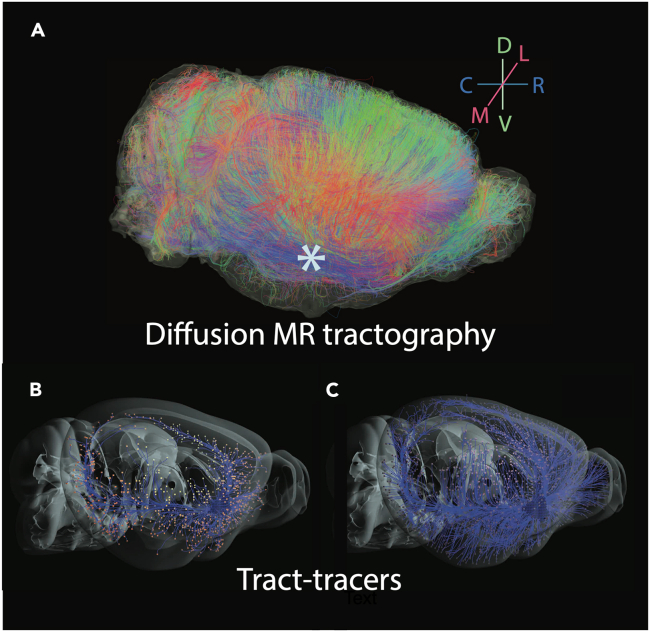
We recommend comparing diffusion MR tractography (A) with tract-tracers (B and C) Here, we found strong concordance in the direction of select fibers in the white matter (asterisk; A) with those from tract-tracers, which involves injecting chemicals in the brain to trace their projections and endpoints (shown in yellow; B, C). The agreement in fiber location and direction across these two methods supports the accuracy of streamline reconstruction. The color coding of diffusion MR tractography is based on the average fiber direction. The color scheme for is shown on the top right corner: red fibers primarily course across the medial (M) to lateral (L) axes, blue fibers primarily course across the rostral (R) to caudal (C) axis, and green fibers primarily course across the dorsal (D) to ventral (V) axis. (A) We set parameters for visualization purposes in this P60 mouse brain. We include fibers that are 1mm in length at minimum, and tracts that start and terminate in a brain mask are included in this P60 brain. Some fibers were skipped to facilitate visualization.

The tractography should be compared with tract-tracers to ensure the reconstructed streamlines are consistent with known patterns of connectivity (Figure 6;^5^^,^^22^). Uncertainties in the accuracy of diffusion MR tractography are particularly prevalent at areas such as at the gray to white matter boundary in mouse cerebral cortex.^23^^,^^24^^,^^25^ Qualitatively comparing tractography with tract-tracers is beneficial for validating streamline plausibility; the Allen Brain Institute has a compendium of tract-tracer information available for visualization.^26^ Tract-tracers label relatively few fibers, but they are the gold standard to study projections.

Quality control step 1: Check for long-range, winding, and abnormal streamline trajectories in well characterized pathways (e.g., corticospinal tract, thalamo-cortical pathway). Ensure that the tractography pattern matches what has been previously reported.

Quality control step 2: Use the Allen Brain Atlas to visualize tract-tracers that are relevant to the pathways of interest. Assess whether the location and orientation of reconstructed fibers in the white matter and ensure these align with those observed from tract-tracers (Figure 6). Tract-tracers may show false positives or negatives. To address this, we recommend evaluating several tract-tracers to compare the consensus from tract-tracers with diffusion MR tractography rather than to focus on a single tract-tracer reconstruction.

***Note:*** Reconstructed tractographies may not resemble patterns observed with tract-tracers. If this is the case, we recommend testing multiple inversions and swaps to ensure the appropriate image orientation is selected to reconstruct biologically plausible pathways (Problem 6).

## Quantification and statistical analysis

There are numerous ways to analyze diffusion MR data and tractography. We outline how we assessed our MRI data in the context of disease and development, but analysis strategy will vary depending on specific research goals. DTI metrics (FA, MD, AD, and RD) convey quantitative diffusivity information about the tissue and act as approximate measures of microstructural integrity. They are sensitive to differences in the magnitude of diffusion, the degree of anisotropy, and the orientation of directional diffusion, providing insight into cytoarchitectural characteristics that are influenced by biological processes such as ischemia, myelination, axon damage, inflammation, and edema.^17^ Group-wise differential analyses or correlation with behavioral, cognitive, or clinical variables can be performed. For example, age is a determining factor for diffusivity metrics and alterations in these relationships may have biological implications. Quantifying tractography, on the other hand, is challenging because there are uncertainties in its accuracy, especially at the grey to white matter boundary. We recommend a classification analysis based on the fiber orientation and overall trajectory of streamlines within the white matter.^7^^,^^10^

1. Quantifying Diffusivity in DSI Studio

We quantified diffusivity metrics from regions of interest (ROIs) based on the Interactive Atlas Viewer from the Allen Brain Atlas. We use this atlas delineations various regions, such as the cerebral cortex, cerebellum, brainstem, and spinal cord.^6^^,^^7^ DSI studio provides information on the mean, minimum, and maximum of each metric, as well as ROI voxel count. We used the diffusivity data extracted from DSI studio to test for regional differences in cerebellar structure between control and spinal muscular atrophy mice. ROI selections may be study specific. For region-wise diffusivity comparisons, we recommend distinguishing white and gray matter because microstructural interpretations are specific to tissue type.^17^

***Note:*** The XP Pen and Wacom display facilitates voxel selection in DSI studio (Table 2).

**Figure 7:**
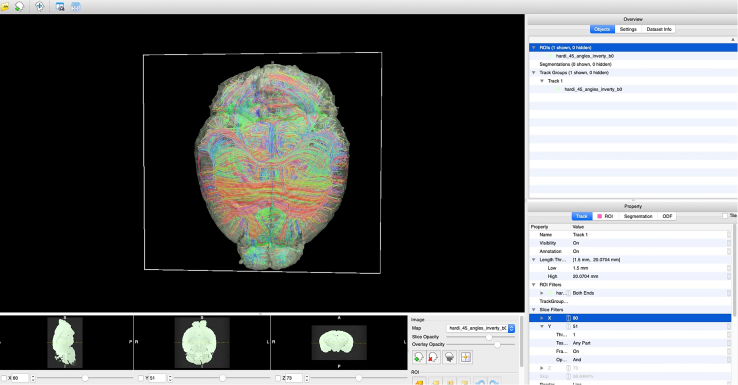
The general user interface shows the tractography and 3D brain image (i.e., a *b*_*0*_ image)

**Figure 8:**
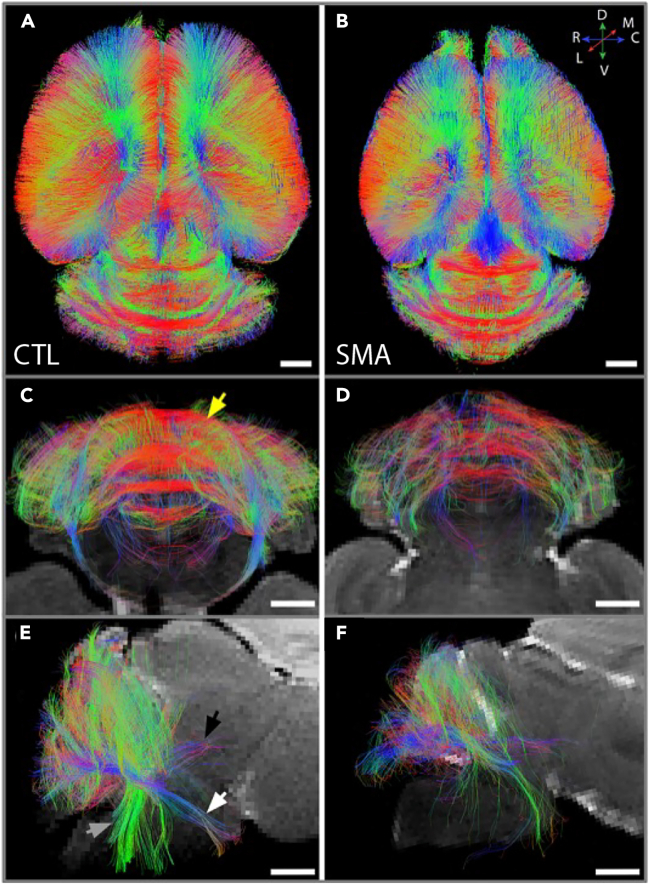
Diffusion MR tractography can be used to compare pathways in mouse models We can use whole-brain and cerebellar tractography in control (CTL; A) and spinal muscular atrophy (SMA; B) mice at postnatal day 12 to detect pathway deficits in this SMA. Close-up views show cerebellar tractography in (C) control and (D) SMA mice. The yellow arrow points to a tract type that varies across groups. Cerebellar tractography in (E) control and (F) SMA mice (see black, gray, and white arrows) differences in tractography between these two groups. This image was previously published.^10^ A legend shows streamline directionality for animal tractography. Abbreviations: L-M: lateral-medial; R-C: rostral-caudal; D-V: dorsal-ventral. Scale bars = 1 mm.
1. Randomly select voxels in the white matter of interest.
2. Visualize fiber orientation to classify and tally the fiber types observed in each voxel
3. Compute the relative number of identified pathway types per sample.
4. Perform an analysis of variance or *t* test to identify whether there are changes in pathway types between groups.

***Note:*** Subsampling analyses can help ensure reproducibility of results. Randomly select a subset of voxels, compute the relative percentage of pathway types, and compare significance tests between subsampled data relative to all data.

2. Qualitative and quantitative tractography analyses in TrackVis
  We used TrackVis to visualize and perform qualitative and quantitative analyses of white matter fibers.^15^ After generating tractography, we used 3D images (e.g., *b*_*0*_, T1, T2 images) of the brain to visualize the underlying anatomy (Figure 7). Figure 8 provides an example of a group-wise comparison of whole-brain and cerebellar tractography in control and spinal muscular atrophy mice at P12.^10^ Perform qualitative assessment of tractography to ensure that streamlines are representing biologically plausible white matter trajectories. Focus on the tractography of well-studied regions, such as the corpus callosum.
  ***Note:*** We recommend integrating diffusion MR tractography with histological information and tract-tracers when studying diffusion MR tractography to validate suspected differences in brain structure.
  a. Open files with Trackvis:
    i. Select *File*->*Open Track.* Select the file with a.trk ending. This opens the tractography file (Problem 8).
    ii. Select *File*-> *Load image.* Select an ANALYZE file with a.nii ending. We used the *b*_*0*_ NiFTI file (generated from diffusion toolkit), which are 3D images to view.
  b. Select Tracks:
    i. When opening the.trk file, a default track group is created with a Y slice filter. Only fibers coursing through the slice are visible (Problem 8).
    ii. Generate ROIs by hand-draw. Right click “Track” on the upper right-hand corner, select a new hand-drawn ROI. Draw ROI on the anatomical images.
      We developed a new approach to quantify connections and types of pathways. This approach relies on voxel-wise manual classification of streamlines. Streamlines were assigned to tract categories based on fiber trajectories and orientations rather than endpoints alone.^7^^,^^10^ For the cerebellum, we categorized cerebellar streamlines as being superior, inferior, or middle as defined by the location in peduncles. For the cerebral cortex, we considered projection endpoints (see Table 3).
      ***Note:*** The choice of tract categories depends on the pathways of interest.

**Table 3:** Examples of pathway classifications in the cerebral cortex^7^^,^^25^

Pathway type	Definitions
Intra-cortico-cortical projection	Projections end their streamline towards the grey matter of the cortex but do not point or extend contralaterally
Cortico-subcortical projection	One projection ends their streamline towards the grey matter of the cortex while another points towards subcortical structures
Corpus callosum	One projection extends towards the corpus callosum
      We made region-to-region connectivity comparisons by calculating the prevalence of various tract types found in ROIs.^7^^,^^10^^,^^25^ This avoids the use of both streamline numbers, which do not necessarily correspond to axon numbers,^26^^,^^27^ and precise streamline termination locations, which can often be erroneous.^23^^,^^24^^,^^27^^,^^28^ We randomly selected voxels to quantify connectivity strength between ROIs.

## Limitations

We provided guidance on how to qualitatively compare diffusion MR tractography with tract-tracers.^29^^,^^30^^,^^31^^,^^32^ These qualitative comparisons are useful to ensure that the steps to reconstruct tractographies were accurately followed and to confirm that tractography reconstructions generally align with tract-tracing results. However, more work is needed to quantitatively compare diffusion MR tractography with tract-tracers so that we may comprehensively compare diffusion MR tractography with tract-tracer results. Tract-tracers and diffusion MR tractography measure properties of the brain at different scales. Integrating information across these scales is a next critical step, which will allow us to distinguish true positives, false positives, and false negatives in diffusion MR tractography.

## Troubleshooting

### Problem 1

When preparing the sample for scanning, air gaps may be present inside the test tube and around the brain. These air bubbles create image artifacts.

### Potential solution

Gently agitate the tube. You may finger flick or tap the tube to remove air bubbles before scanning. Fully submerge the brain with no air bubbles in or around the brain for optimal image.

### Problem 2

You are not able to scan the brain.

### Potential solution

Verify that the coils are functional before scanning. Coil failure is a common issue.

### Problem 3

The image quality is irregular. This irregularity appears in regions near the brain’s edge.

### Potential solution

Irregularity in brain image is likely because some of the brain is outside the coil coverage. In coil-based imaging setups, the signal drops off sharply when tissue lies outside or even is near the boundary of the coil’s effective sensitivity region. Verify that the brain is placed within the coil’s’ coverage. Ideally, place the brain at the center of the coil.

### Problem 4

The brain MRI scan appears damaged.

### Potential solution

Some scan images may be of poor quality because of air bubbles, the brain is damaged, or the brain lies outside the coil. In those cases, it may be best to omit those images for subsequent analyses.

### Problem 5

Files may become corrupt.

### Potential solution

Reupload the source data to the data storage center. Files may become corrupt during upload.

### Problem 6

The computer hardware is unable to run DSI studio.

### Potential solution

Use the recommended download from DSI Studio based on actual hardware capabilities. Avoid opening multiple image viewers at once. DSI Studio offers multiple software versions based on operating systems (Windows, MacOS, Ubuntu, etc.) and hardware (GPU vs CPU). Our protocol used the “Windows with a NVIDIA GPU” version. This is based on the hardware limitation of the computer.

### Problem 7

The tractography reconstruction appears biologically implausible. Your tractography may appear flipped and it may not align with observations from tract-tracers.

### Potential solution

You may need to reconstruct streamlines with different MRI coordinates to find the appropriate tractography.

### Problem 8

The TrackVis program might freeze or crash.

### Potential solution

There are a number of strategies to reduce the likelihood the program crashes. Use plain lines as render type for tracks, avoid opening multiple windows, and restrict the number of objects you are working with (e.g., track groups). Turn off the “Force Render” option. Also, avoid using multiple intensive cpu/graphics, which may consume RAM.

## Resource availability

### Lead contact

Requests for further information and resources should be directed to and will be fulfilled by the lead contact, Christine Charvet (charvetcj@gmail.com).

### Technical contact

Technical questions on executing this protocol should be directed to and will be answered by the technical contact, Nicholas Cottam (nickcottam4@gmail.com).

### Materials availability

DSI Studio is free for academic users under Attribution-NonCommercial-ShareAlike 4.0 International License (CC BY-NC-SA 4.0). DSI Studio is available for download at https://dsi-studio.labsolver.org/download.html.

The version used for our protocol is “Chen” 2024, and it is currently at version “Hou” 2025. For a Commercial License, refer to the DSI Studio website for commercial licensing and application.

TrackVis and Diffusion Toolkit are free for users after registration (https://trackvis.org/dtk/).

Bruker materials can be obtained at https://www.bruker.com/en/products-and-solutions/preclinical-imaging/mri.html.

Bruker Biospec 9.4 T MRI information can be found at https://www.bruker.com/en/products-and-solutions/preclinical-imaging/mri/biospec.html.

Bruker Paravision Software information can be found at https://www.bruker.com/en/products-and-solutions/preclinical-imaging/paravision-360.html.

Bruker Coil availability and information can be found at https://www.bruker.com/en/products-and-solutions/preclinical-imaging/mri/mri-rf-coils.html.

Additional information can be found at https://www.bruker.com/en/products-and-solutions/preclinical-imaging/mri/mri-rf-coils/mri-rf-coils-technical-details.html.

### Data and code availability

• Diffusion MR scans and R scripts are available as Dryad datasets.
• Cottam N, Ofori K, Stoll K, Madison B, Rogge J, Hekmatyar K, Sun J, Charvet CJ. (2025). Data from: From circuits to lifespan: Translating mouse and human timelines with neuroimaging based tractography [Dataset]. Dryad. https://doi.org/10.5061/dryad.8pk0p2nzt.
• Charvet C (2022). Tracing pathways from high-resolution tractography, transcription, and temporal dimensions [Dataset]. Dryad. https://doi.org/10.5061/dryad.9w0vt4bg9.

## Acknowledgments

This work was supported by the 10.13039/100009633Eunice Kennedy Shriver National Institute of Child Health and Human Development Grants 1R21-HD-101964-01A1, 7R21-HD101964-02 (to C.J.C.), and T32HD007489 (to N.C.C.); National Institutes of Neurological Disorders R15NS120154 (to J.S.); Institutional Development Award (IDeA) Networks of Biomedical Research Excellence (INBRE) Pilot Grant P20-GM-103446 from the 10.13039/100000057National Institute of General Medical Sciences (NIGMS; to C.J.C.); 10.13039/100000057NIGMS Core Center Access Award P20-GM-103446; and the Centers of Biomedical Research Excellence Grant 5P20-GM-103653 for research at Delaware State University. Opinions are not necessarily those of the 10.13039/100000002National Institutes of Health. Mouse brain scanning was completed at the University of Delaware’s Center for Biomedical and Brain Imaging (CBBI). Some images were made with BioRender. We acknowledge Ibrahim Malik for designing the 3D brain mold and providing the supplementary file.

## Author contributions

All authors participated in the design of the protocol and writing of this manuscript. Conceptual design of the protocol was led by J.S., N.C.C., and C.J.C. Conceptual design of the analyses of MRI images were led by N.C.C., J.S., K.T.S., and C.J.C. Writing, editing, and figure preparation were led by N.C.C., K.T.S., J.S., and C.J.C.

## Declaration of interests

The authors declare no competing interests.

## Declaration of generative AI and AI-assisted technologies in the writing process

During the preparation of this work, we used ChatGPT in select sentences to edit text for flow and grammar. After using this tool/service, we reviewed and edited the content as needed and we take full responsibility for the content of the published article.

## Supplemental information

File S1. Data to generate the 3D brain mold
